# Supplementary material for: Ecdysone coordinates plastic growth with robust pattern in the developing wing
Source: eLife. 2022 Mar 9;11:e72666. doi: 10.7554/eLife.72666 (PMC8947767; doi:10.7554/eLife.72666)
Supplement: Supplementary file 1. [file elife-72666-supp1.docx]

**Supplementary File 1a:** Comparing the Gompertz growth curve parameters for wing imaginal discs from PGX and control (+>Grim and phm>+) larvae.

| Genotype/Condition | Asymptote (*a*)^A^ | *F*^B^ | Displacement along x (*b*)^A^ | *F*^B^ | Growth rate (*g*)^A^ | *F*^B^ |
| --- | --- | --- | --- | --- | --- | --- |
| PGX | 10.53 (10.45–10.65) | 52.62*** | 0.11 (0.10–0.13) | 68.91*** | 0.90 (0.85–0.94) | 5.45* |
| Control | 11.57 (11.42-11.74) |  | 0.22 (0.21–0.24) |  | 0.95 (0.94–0.95) |  |

^A^ Parameter values are for a Gompertz model $y = a\cdot e^{-b\cdot e^{-g\cdot x}},$where x is disc size and y is Achaete pattern. Values in parentheses are 95% confidence intervals.

^B^ *F*-test of whether parameter value differs between genotype/condition. * *P* value < 0.05, *** *P* value < 0.0001.

**Supplementary File 1b:** Comparing the Gompertz curve parameters of Achaete patterning against time for wing imaginal discs from PGX and control (+>Grim and phm>+) larvae.

| Genotype/Condition | Asymptote (*a*)^A^ | *F*^B^ | Displacement along x (*b*)^A^ | *F*^B^ | Growth rate (*g*)^A^ | *F*^B^ |
| --- | --- | --- | --- | --- | --- | --- |
| PGX | 3.98 (3.56–4.41) | 12.62*** | 1.03(0.84–1.21) | 12.05*** | 0.045 (0.029-0.063) | 0.56 |
| Control | 6.34 (6.20–7.08) |  | 1.64 (1.42–1.87) |  | 0.074  (0.059-0.090) |  |

^A^ Parameter values are for a Gompertz model $y = a\cdot e^{-b\cdot e^{-g\cdot x}},$where x is disc size and y is Achaete pattern. Values in parentheses are 95% confidence intervals.

^B^ *F*-test of whether parameter value differs between genotype/condition. * *P* value < 0.05, *** *P* value < 0.0001.

**Supplementary File 1c:** Comparing the linear Senseless patterning parameters for wing imaginal discs from PGX and control (+>Grim and phm>+) larvae.

| Genotype | Intercept (*a*)^A^ | F^B^ | Slope (*b*)^A^ | F^B^ |
| --- | --- | --- | --- | --- |
| PGX | 1.25 (0.94–1.55) | 25.89*** | 0.01(0.00–0.02) | 583.26*** |
| Control | 0.46 (0.21–0.72) |  | 0.13 (10.12–0.14) |  |

^A^ Parameter values are for a linear model *y = B + Ax*, where x is disc size and y is Senseless pattern. Values in parentheses are 95% confidence intervals.

^B^ *F*-test of whether parameter value differs between genotype/condition. * *P* value < 0.05, *** *P* value < 0.0001.

**Supplementary File 1d:** The effect of supplementing 20-hydroxyecdysone (20E) on wing disc size, Achaete stage, and Senseless stage in control and PGX larvae

| Factors | F value – Disc Size | F value – Achaete Stage | F value – Senseless Stage |
| --- | --- | --- | --- |
| Genotype^A^ | 162.78*** | 210.49*** | 265.06*** |
| 20E Treatment^B^ | 135.95*** | 56.38*** | 129.21*** |
| Genotype * Treatment | 72.87*** | 120.26*** | 163.72*** |

^A^ Genotypes include PGX and control larvae.

^B^ 20E Treatment includes supplementation with 0.15 mg/ml of 20E or the same volume of ethanol

*** *P* value < 0.001

**Supplementary File 1e:** The effect of supplementing 20-hydroxyecdysone (20E) on wing disc size, Achaete stage, and Senseless stage in control and PGX larvae reared on starvation medium or on fly food.

| Factors | F value – Disc Size | F value – Achaete Stage | F value – Senseless Stage |
| --- | --- | --- | --- |
| Genotype^A^ | 1.15 | 0.48 | 0.00 |
| 20E + Diet Treatment^B^ | 214.20*** | 358.80*** | 117.22*** |
| Genotype * 20E + Diet Treatment | 28.86*** | 99.76*** | 72.08*** |

^A^ Genotypes include PGX and control larvae.

^B^ 20E + Diet Treatment includes starved + 0.15 mg/ml 20E, starved + ethanol, and fed

*** *P* value < 0.001

**Supplementary File 1f:** Comparing the parameters of the linear relationship for Achaete pattern against (log) disc size between phm > InR and their parental control line (+ > InR), and between P0206 > PTEN and their parental control line (+ > PTEN).

| Genotype/  Condition | Intercept (*a*) ^A^ | *F*^B^ | Slope (*b*) ^A^ | *F*^B^ |
| --- | --- | --- | --- | --- |
| phm>InR | -14.99 (-17.32–-12.65) | 22.42*** | 1.93 (1.70-2.15) | 660.67** |
| P0206>PTEN | - 12.23(-13.72–-10.75) |  | 1.61 (1.44–1.78) |  |
| Control | -16.40 (-18.45–-14.23) |  | 1.97 (1.83-2.10) |  |

^A^ Parameter values are for a linear model *y = B + Ax*, where x is disc size and y is Achaete pattern. Values in parentheses are 95% confidence intervals.

^B^ *F*-test for when the parameter value differs between genotype/condition. ^NS^ *P* value > 0.05, **P* value < 0.05, **P* value < 0.01, *** *P* value < 0.001.

**Supplementary File 1g:** Comparing the parameters of the logistic relationship for Senseless pattern against (log) disc size between phm > InR and their parental control line (+ > InR), and between P0206 > PTEN and their parental control line (+ > PTEN).

| Genotype/  Condition | Minimum (*a*)^A^ | F^B^ | Maximum (*b*)^A^ | F^B^ | Point of inflection (*c*)^A^ | F^B^ | Logistic Growth rate (*d*)^A^ | F^B^ |
| --- | --- | --- | --- | --- | --- | --- | --- | --- |
| Phm>InR | 0.92 (0.32 – 1.52) | 0.12^NS^ | 7.13 (5.72–8.54) | 3.61* | 10.42 (10.14–10.69) | 6.94** | 2.13 (1.40 – 4.45) | 5.89** |
| P0206>PTEN | 1.00 (0.73-1.27) |  | 7.15 (6.31–8.00) |  | 10.97 (10.84–11..10) |  | 4.65 (3.31–7.84) |  |
| Control | 1.04 (0.80-1.29) |  | 8.91 (7.08–10.73) |  | 11.22 (10.99–11.45) |  | 2.39 (1.86–3.35) |  |

^A^ Parameter values for the four-parameter logistic model $y=a+\left( b-a \right)/\left( 1+e^{d\left( c-x \right)} \right)$ where x is disc size and y is Senseless pattern. Values in parentheses are Bonferroni-corrected 95% confidence intervals.

^B^ *F*-test for when the parameter value differs between genotype/condition. ^NS^ *P* value > 0.05, **P* value < 0.05, **P* value < 0.01, *** *P* value < 0.001.

**Supplementary File 1h** Effect of 20E supplementation on titres of ecdysteroids in control (phm > + and + > grim) and PGX larvae under starved and fed conditions.

| Factor | df | F value | *P* |
| --- | --- | --- | --- |
| 20E (poly, 2) | 1 | 69.60 | <0.001 |
| Food | 2 | 59.14 | <0.001 |
| Genotype Group | 1 | 6.05 | 0.015 |
| 20E (poly, 2): Food | 1 | 6.14 | 0.003 |
| 20E (poly, 2): Genotype Group | 2 | 7.32 | 0.001 |
| Food: Genotype Group | 2 | 2.53 | 0.113 |
| 20E (poly, 2): Food: Genotype Group | 2 | 6.21 | 0.003 |

**Supplementary File 1i:** Effect of diet type on wing disc growth in PGX and control (phm > + and + > grim) larvae under starved and fed conditions without 20E.

| Factor | df | *F* value | *P* |
| --- | --- | --- | --- |
| Genotype | 1 | 1.4 | 0.24 |
| Food | 1 | 278.5 | <0.001 |
| Time^2^ | 2 | 202.5 | <0.001 |
| Genotype: Food | 1 | 8.7 | 0.003 |
| Genotype: Time^2^ | 2 | 13.2 | <0.001 |
| Food: Time^2^ | 2 | 98.7 | <0.001 |
| Genotype: Food: Time^2^ | 2 | 5.31 | 0.005 |

**Supplementary File 1j:** Effect of 20E concentration on growth of the wing imaginal disc in starved and fed PGX larvae.

|  | Starved | | | | Fed | | | |
| --- | --- | --- | --- | --- | --- | --- | --- | --- |
| Factor^A^ | SS | df | *F* | *P* | SS | df | *F* | *P* |
| 20E | 1.5 | 1 | 27.3 | <0.001 | 0 | 1 | 1.35E-01 | 0.7137 |
| Disc Age^2^ | 7.2 | 2 | 64.6 | <0.001 | 36.6 | 2 | 29.8 | <0.001 |
| 20E: Disc Age^2^ | 1 | 2 | 8.88 | <0.001 | 0 | 2 | 4.01E-01 | 0.6697 |
| Residuals | 22.3 | 403 |  |  | 27.8 | 453 |  |  |

^A^ Factors were fit using an orthogonal polynomial regression.

**Supplementary File 1k:** Comparisons of model fit for linear rates of wing disc growth, Achaete patterning, and Senseless patterning across 20E concentrations in fed and starved larvae.

.

|  |  | Wing growth rate - starved | | Achaete patterning rate - fed | | Achaete patterning rate - starved | | Senseless patterning rate - fed | | Senseless patterning rate - fed | |
| --- | --- | --- | --- | --- | --- | --- | --- | --- | --- | --- | --- |
|  | df | AIC | BIC | AIC | BIC | AIC | BIC | AIC | BIC | AIC | BIC |
| Michaelis Menten^A^ | 4 | **-43.67** | **-44.50** | -22.72 | -23.55 | -18.69 | -19.52 | -29.88 | -30.71 | -29.64 | -30.48 |
| Three parameter logistic^B^ | 4 | -30.72 | -31.55 | -25.28 | -26.12 | -29.83 | -30.66 | -40.84 | -41.68 | **-35.09** | **-35.93** |
| Four parameter logistic^C^ | 5 | -41.73 | -42.77 | **-32.99** | **-34.03** | **-46.67** | **-47.71** | **-48.52** | **-49.56** | -33.37 | -34.41 |

^A^ Michaelis Menten function: *y =  c + (d-c)/(1+(b/x))*, where *c* is *y*at *x=*0*, d* = *y[max]*, and*b* is *x* where *y* is halfway between *c*and *d*.

^B^ Three-parameter log-logistic function: *y = d/(1+e^(b(log(x)-log(a))^)*, where *d* = *y[max]*, *b* is the rate of increase, and *a* is the inflection point. Note, in the three-parameter log-logistic function, c = 0.

^C^ Four-parameter log-logistic function: *y = c + (d-c)/(1+e^(b(log(x)-log(a))^)*, where *c* is *y*at *x=*0*, d* = *y[max], b* is the rate of increase, and *a* is the inflection point.

The selected model has the lowest AIC and BIC values, and is in bold.

**Supplementary File 1l:** Effect of 20E concentration on Achaete patterning stage in the wing imaginal disc in fed and starved PGX larvae.

|  | Fed | | | | Starved | | | |
| --- | --- | --- | --- | --- | --- | --- | --- | --- |
| Factor^A^ | SS | df | *F* | *P* | SS | df | *F* | *P* |
| 20E | 124.9 | 5 | 72.65 | <0.001 | 152.7 | 5 | 75.82 | <0.001 |
| Disc Age^2^ | 4.7 | 2 | 6.78 | 0.0013 | 1.4 | 2 | 1.70 | 0.18 |
| 20E: Disc Age^2^ | 108.3 | 10 | 31.48 | <0.001 | 178.0 | 10 | 44.20 | <0.001 |
| Residuals | 151.7 | 441 |  |  | 157.48 | 391 |  |  |

^A^ Factors were fit using an orthogonal polynomial regression.

**Supplementary File 1m** Effect of 20E concentration on Senseless patterning stage in the wing imaginal disc in fed and starved PGX larvae.

|  | Fed | | | | Starved | | | |
| --- | --- | --- | --- | --- | --- | --- | --- | --- |
| Factor^A^ | SS | df | *F* | *P* | SS | df | *F* | *P* |
| 20E | 34.3 | 5 | 44.55 | <0.001 | 56.7 | 5 | 55.08 | <0.001 |
| Disc Age^2^ | 0.72 | 2 | 2.34 | 0.097 | 0 | 2 | 0 | 1 |
| 20E: Disc Age^2^ | 37.4 | 10 | 24.28 | <0.001 | 70.45 | 10 | 27.36 | <0.001 |
| Residuals | 67.84 | 441 |  |  | 100.7 | 391 |  |  |

^A^ Factors were fit using an orthogonal polynomial regression.
